# Reaching People With Disabilities in Underserved Areas Through Digital Interventions: Systematic Review

**DOI:** 10.2196/12981

**Published:** 2019-10-25

**Authors:** Leming Zhou, Bambang Parmanto

**Affiliations:** 1 Department of Health Information Management University of Pittsburgh Pittsburgh, PA United States

**Keywords:** systematic review, digital intervention, electronic intervention, e-intervention, underserved area, disability, telemedicine, telerehabilitation, eHealth, digital health

## Abstract

**Background:**

People with disabilities need rehabilitation interventions to improve their physical functioning, mental status, and quality of life. Many rehabilitation interventions can be delivered electronically ("digitally") via telehealth systems. For people with disabilities in underserved areas, electronically delivered rehabilitation interventions may be the only feasible service available for them.

**Objective:**

The objective of this study was to evaluate the current status of digital interventions for people with disabilities in remote and underserved areas.

**Methods:**

A systematic review was conducted on this topic. Keyword searches in multiple databases (PubMed, CINAHL, and Inspec) were performed to collect articles published in this field. The obtained articles were selected based on our selection criteria. Of the 198 identified articles, 16 duplicates were removed. After a review of the titles and abstracts of the remaining articles, 165 were determined to be irrelevant to this study and were therefore removed. The full texts of the remaining 17 articles were reviewed, and 6 of these articles were removed as being irrelevant to this study. The 11 articles remaining were discussed and summarized by 2 reviewers.

**Results:**

These 11 studies cover a few types of disabilities, such as developmental disabilities and mobility impairments as well as several types of disability-causing disorders such as stroke, multiple sclerosis, traumatic brain injury, and facio-scapulo-humeral muscular dystrophy. Most of these studies were small-scale case studies and relatively larger-scale cohort studies; the project evaluation methods were mainly pre-post comparison, questionnaires, and interviews. A few studies also performed objective assessment of functional improvement. The intervention technology was mainly videoconferencing. Moreover, 10 of these studies were for people with disabilities in rural areas and 1 was for people in urban communities.

**Conclusions:**

A small number of small-scale studies have been conducted on digital interventions for people with disabilities in underserved areas. Although the results reported in these studies were mostly positive, they are not sufficient to prove the effectiveness of telehealth-based digital intervention in improving the situation among people with disabilities because of the small sample sizes and lack of randomized controlled trials.

## Introduction

### Background

Advancement in health care technologies has contributed to the decline of mortality in the United States in recent years. Many people survive diseases that used to have a high mortality rate [1]. However, surviving a severe disease is often not the end of the story but the beginning of a life filled with many serious challenges. We use 2 specific examples to demonstrate these challenges.

In the United States, although stroke is still one of the top 10 leading causes of death [2] and stroke prevalence has increased in recent years because of the increase in the aging population [3], the actual number of deaths caused by stroke declined by 2.3% from 2005 to 2015 [1]. There are approximately 6.8 million stroke survivors in the United States [1], but only 10% of stroke survivors recover almost completely. Nearly 65% of stroke patients experience significant or permanent disabilities such as paralysis, urinary incontinence, aphasia, and cognitive disability. In all, about 3% of adults in the United States claim that they were disabled because of stroke [1]. These disabled stroke survivors need long-term rehabilitation services to relearn the skills lost in the stroke attack. Previous research has reported that people who participate in focused poststroke rehabilitation programs perform better than most people who do not undergo poststroke rehabilitation [4-6]. Depending on the complications after stroke, the rehabilitation plan for each patient must be personalized, and the stroke rehabilitation may involve a variety of specialists for a long period.

Traumatic brain injury (TBI) is another leading cause of death and long-term disability. It is estimated that 1.7 million TBIs occur in the United States annually, which contribute to approximately 30% of all injury deaths. Advances in trauma care have resulted in increases in the number of survivors of TBI in recent years. In the United States, there are approximately 5.3 million individuals living with disabilities caused by TBI [7]. For TBI survivors, there may be physical, behavioral, and psychological alterations that require extensive rehabilitation services over a long period to restore skill [7,8].

There are also other causes of disability that may not be as severe as stroke or TBI but which also present similar challenges. Multiple sclerosis (MS) is a neurological disorder that leads to several conditions and disabilities that limit several daily life activities [9,10]. Some of these conditions are associated with low rates of physical activity, including fatigue, weakness, falls, and depression [11,12]. Previous studies have demonstrated that exercise and physical activity can reduce symptoms, increase physical health, and improve quality of life for people with MS [13].

Developmental disabilities (DDs) are a group of conditions such as intellectual disability, learning disability, cerebral palsy (CP), hearing loss, autism spectrum disorder, attention deficit hyperactivity disorder, and other developmental delays because of physical, language, learning, or behavior impairments that happened as an infant or during development as a fetus. These conditions may impact children’s daily functioning and usually last for a lifetime [14]. Recent estimates suggest that about 15% of children aged between 3 and 17 years have one or more development disability (DD) in the United States [15]. Early intervention (EI) is the service and support to children with DD and their families, for example, speech and language therapy (SLT), physical therapy (PT), and other types of services, based on the conditions and needs of the children and their family. EI can have a significant impact on a child’s ability to learn new skills and can increase success in life and school. However, EI is more effective if delivered in the natural environment of babies and from the very early stages of childhood, which is challenging for families who live in rural communities.

For many survivors of stroke or TBI and people with MS or DD, the common need is long-term intervention. A comprehensive, coordinated rehabilitation program can reduce secondary complications and improve functional outcomes. Many rehabilitation protocols require multidisciplinary, high-intensity therapy sessions multiple times a week, for weeks, months, or even years [16]. Some rehabilitation services need to be delivered at a very specific time and frequency. All of these requirements are difficult to meet for people in the rural and remote areas as there are extraordinary physical, financial, and logistical hardships.

Specifically, according to the US Census Bureau, approximately 20% of the US population lives in rural areas [17]; however, less than 8% of the nation’s physicians are practicing in rural areas. The majority of these physicians are in primary care such as family practice, general internal medicine, and pediatrics [18,19]. In other words, receiving regular health care services in rural areas is challenging, it is even harder for people with disabilities in rural areas to access highly qualified specialists for poststroke, post-TBI, or EI rehabilitation services [18,20-22]. Traveling to major cities and seeking the desired intervention costs a lot in terms of money and time, which can be a very heavy burden for family members of the patients.

People with disabilities in poor urban communities face different challenges in terms of receiving such services. Although geographical distance may not be such a huge issue in terms of accessing health care services, difficulties with transportation, dependence on caregivers, low health literacy, and lower socioeconomic status still create significant challenges in terms of access to high-quality health care for people with disabilities in poor urban communities [23]. For this reason, in this study, both rural areas and poor urban communities are referred to as *underserved areas*.

### Digital Interventions

Telehealth may be a viable approach for the delivery of interventions to people with disabilities in underserved areas [24]. The concept of telehealth has been discussed since the 1960s, but then it consisted mainly of using telephones to provide communication between patients and health care providers. Since the 1990s, the availability of the internet has made it possible to use multiple information and communication technologies (ICTs) to deliver digital interventions via telehealth. Further development of ICT and the high penetration of broadband connection at home in the 2000s have made it possible for people with disabilities and health care providers to communicate more easily via videoconferencing (VC). A national survey in June 2019 indicated that 90% of American adults used the internet, 73% of American adults used broadband connections at home, and 63% of rural American homes are connected to the internet, and 17% of US adults only use smartphones to access the internet [25]. According to a national survey performed by Pew Research Center, also in June 2019, the adoption of smartphone and other smart mobile devices has increased dramatically as well in recent years. In 2011, 35% Americans owned smartphones, whereas in 2019, the rate of smartphone ownership is 81% overall. In rural areas, the smartphone ownership rate is 71% [26]. In other words, the improvement of ICT in recent years and the penetration of the internet and mobile devices make it easier than ever to conduct VC, which is the foundation of many telehealth systems. This situation makes telehealth a feasible approach for delivering digital intervention to people with disabilities in underserved areas.

Telehealth has been used to provide assistive technology assessment [27,28], diagnostic evaluations [29,30], assessment and therapy services [31], and consultation opportunities for practitioners and people with disabilities in rural communities [32]. The benefits of telehealth include access to high-quality care, reduced travel time and costs, and increased collaboration among health care providers [29]. Previous studies have indicated that telehealth is a potentially efficient and effective alternative to hospital-based care to deliver patient-satisfying health care services [33,34].

Telehealth enables therapists to deliver rehabilitative services to patients who cannot access health care providers because of physical, financial, and logistical barriers [35]. In recent years, as technologies have become ubiquitous and costs have declined, it has become easier to support the use of telehealth. As a result, research on telehealth has begun to switch from pilot and case studies to validity and reliability of interventions delivered via telehealth.

There are many ways to categorize telehealth services. In this study, we categorize them into three groups based on the specific technologies used in intervention delivery:

1. Regular phone calls, short message service text messages, interactive voice responses (IVRs), and emails
2. VC using technologies such as Skype and video phone
3. Mobile health apps in telehealth practice

Phone calls, text messages, IVRs, and emails can be useful for encouragement and reminders if patients and caregivers are already familiar with the procedure of the intervention. If that is not the case, these methods cannot deliver new and personalized interventions to people with disabilities in underserved areas. Therefore, in this review, we will not include studies using the technologies in the first category.

### Objectives

In this study, our goal was to determine the current status of digital interventions delivered to people with disabilities via telehealth in underserved areas; more specifically, we investigated the type of disabilities covered in recent research studies, the number of people with disabilities involved in those studies, the technologies used in the studies, and the outcomes of those studies.

Although a few similar systematic reviews have been conducted in previous years, the covered studies were not high in quality and the results were not generalizable. In this study, we want to determine whether the situation has improved in recent years in terms of telehealth research on those in underserved area.

## Methods

### Overview

This systematic review was conducted and reported according to the Preferred Reporting Items for Systematic Review and Meta-Analysis guidelines [36]. Methods of the review process and eligibility criteria were established in advance, and the preliminary results were presented orally at the Rehabilitation Engineering and Assistive Technology Society of North America annual conference in 2018.

### Literature Search

The keywords used in this study were “(Telehealth OR mHealth OR telerehabilitation OR eHealth OR telemedicine) AND (disability or impairment) AND (underserved OR rural).” In June 2018, the keyword searches were first performed only in PubMed to obtain a general idea of the number of studies in this area. When only “telehealth” was used in the keyword search, there were 27,900 results from PubMed. When “telehealth AND disability” was used, the returned number of studies was 422. When “telehealth AND disability AND (underserved OR rural)” was used, the obtained number of studies was only 64. The numbers of results were similar when other similar keyword combinations were used in PubMed.

In June 2018, the keyword searches were performed for peer-reviewed journal and conference research articles in 3 bibliographic databases: PubMed, CINAHL, and Inspec, without any year restriction. In total, there were 198 articles obtained from the 3 databases using the keyword searches. Moreover, 16 articles were determined to be duplicates and, therefore, were removed from the article list immediately. The studies described in this systematic review were selected from the remaining 182 articles according to the selection criteria given below.

### Selection Criteria

#### Publication Year

During the literature search using keywords, there was no limit on the year of the publication. However, as digital interventions via telehealth became widely available only after 2000, the study purpose itself limited the publication period to between 2000 and 2018. There was no limit on the age of the patients. The language of the selected articles had to be English. The articles had to be research papers published at conferences or in journals. Reviews, abstracts, editorials, workshop summaries, perspectives, opinions, diagnosis methods, and study protocols were excluded. The study could have been performed in any country.

#### Population

The population was patients with disabilities (eg, developmental, cognitive, vision, intellectual, and mobility impairments, as well as impairments caused by problems such as TBI, stroke, autism, spinal cord injury, CP, MS, and spina bifida) in underserved areas who participated in telehealth-based digital intervention studies before June 2018. Studies about health care providers who received training or offered teleconsultation were not included in this study.

#### Intervention

The intervention had to be delivered *digitally* via ICTs such as VC-based intervention on mobility for people who had experienced acute stroke. Other examples of interventions are speech language therapy for improving patients’ language ability, occupational therapy (OT) for enhancing participation ability, and psychotherapy for managing stress or depression. If the intervention was delivered only via regular telephone without any video component, email, or IVR, the article was removed from this study. If the article was purely about a telehealth IT system development or a patient condition assessment or monitoring (no intervention), it was removed as well. The setting of the intervention could be a home, nursing home, or clinic in an underserved area.

#### Comparator

The comparator could be either face-to-face intervention or any other control intervention. Articles with no comparison were also included. After all, the purpose of this study was not to determine whether telehealth-based digital interventions are as good as face-to-face interventions but to determine the current status of delivering telehealth-based digital interventions to people with disabilities in underserved areas.

#### Outcomes

The outcomes of studies that were considered were as follows: participants’ satisfaction with the digital intervention, functional improvement in physical and mental aspects, travel time and cost, and general quality of life improvement.

#### Study Design

The eligible study designs were quantitative, qualitative, and mixed-method studies that explored the outcomes of a telehealth-based digital intervention. Case studies and pilot studies were included because it was possible that they could enable us to understand the change in status over time.

### Study Selection

EndNote X7 (Clarivate Analytics) was utilized to manage the articles and collect data from these articles. The selection of the studies was conducted in 3 rounds. In the first round, duplicates were removed from the study list. In the second round, 2 reviewers (LZ and BP) independently reviewed titles and abstracts against the selection criteria, and disagreements were resolved via multiple discussions. In the third round, both reviewers went through the full texts of the remaining articles and made further selection according to the selection criteria.

### Quality Assessment

It was important to assess the quality of the selected studies. The quality criteria were used to verify that the selected studies are relevant to this study and the selected studies themselves were methodologically solid [37]. The 11 selected studies were evaluated with regard to the study purpose, literature review, methodology, results obtained, risk of biases in terms of sampling, measurement and intervention, and the conclusion. For this purpose, the quality of these 11 selected studies was evaluated using a modified version of the critical review form created by researchers at McMaster University [38,39]. More specifically, the 10 questions in Textbox 1 were used to evaluate the quality of these studies. If the answer to a question was yes, the score was 1, otherwise the score was 0. Therefore, the maximum quality score that each study could obtain was 10. If the quality score of a study was less than 6, it was removed from the study.

**Questions used for quality assessment selected from the McMaster University critical review form for quantitative studies.**

To perform quality assessment on papers, the following 10 questions can be used:

1. Was the purpose stated clearly?

2. Was relevant background literature reviewed?

3a. Was the sample described in detail?

3b. Was sample size justified?

4. Were the outcome measures reliable?

5. Intervention was described in detail?

6a. Results were reported in terms of statistical significance?

6b. Were the analysis methods appropriate?

6c. Clinical importance was reported?

7. Conclusions were appropriate given study methods and results?

### Data Extraction and Synthesis

Two reviewers (LZ and BP) extracted data from the 11 articles that were found to meet the inclusion criteria. A standardized form was used for data extraction. Data items on the extraction form include the following: first author’s name; publication year; journal or conference name; disability or disability-causing disease; underserved area; sample size; participants’ age, sex, and race; study design; duration of intervention; intervention; telehealth technology (store-and-forward, teleconferencing, mobile phone app, or other approach); comparator (if applicable); outcome measures; study results; and location of the study (country or state).

## Results

### Study Selection

**Figure 1:**
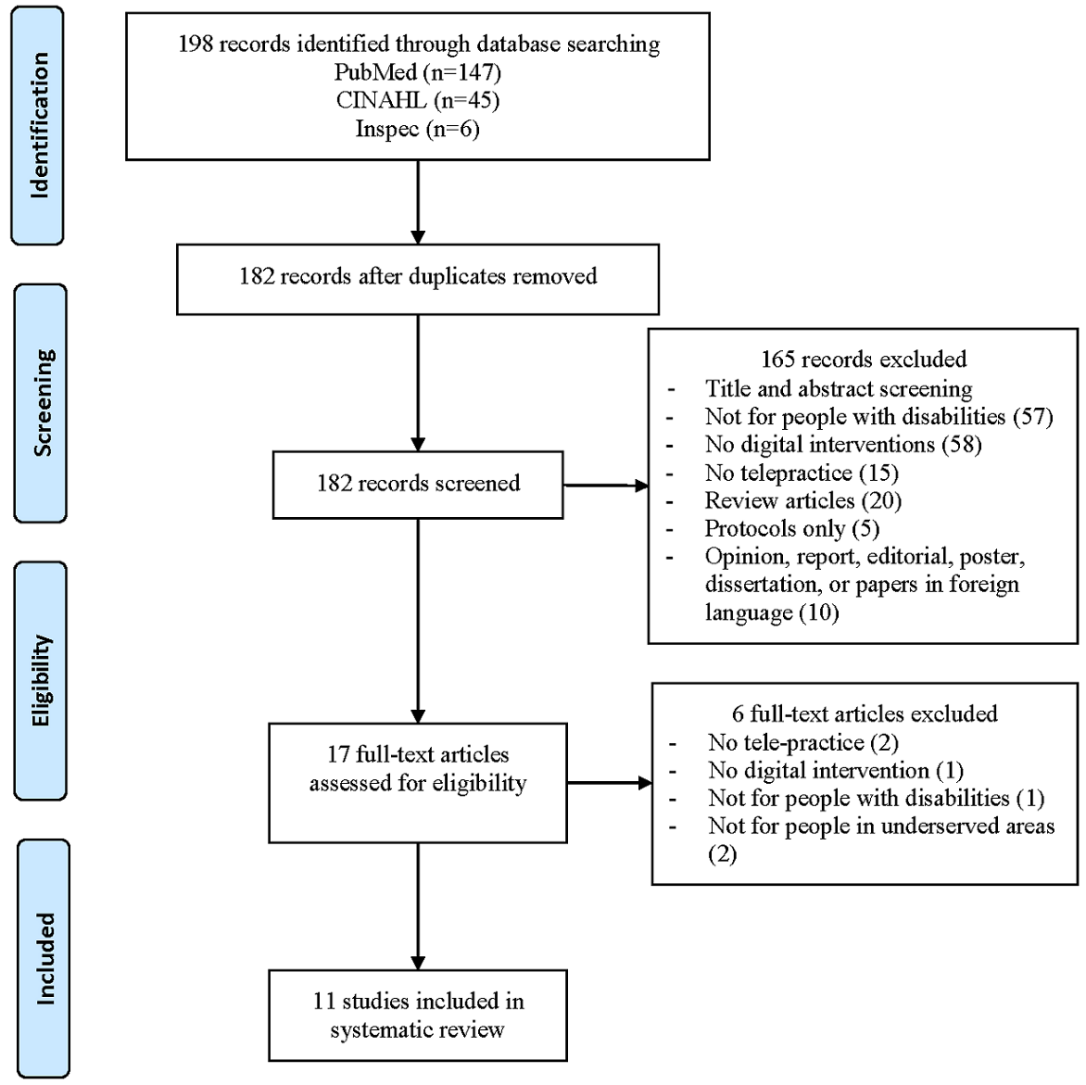
Flowchart of the study selection.

In the first round of the study selection, 16 duplicates were removed from the study list. In the second round, 165 articles were removed from the study list because they were an opinion paper (1), published in foreign language (2), a dissertation (2), an editorial (3), not for people with disabilities (57), did not include any digital interventions (58), did not use telehealth practice (15), a poster (1), a protocol (5), a report (1), or a review article (20). Each count here is only for a violation of one item in the selection criteria to avoid double count. In the third round, 6 articles were removed from the remaining 17 articles because they did not include telepractice (2), did not have any intervention (1), were not for people in underserved areas (2), or were not for people with disabilities (1). Therefore, at the end of the study selection, a total of 11 articles remained. A flowchart for this study selection is shown in Figure 1.

### Quality Assessment

The quality assessment results shown in Table 1 [40-50] illustrate that these 11 studies met the quality criteria for being included in this systematic review. One common problem of these studies is that none of them justified their sample size (item 3b in Textbox 1). The other common problem in more than half of these studies (7/11, 64%) is that authors did not report their results in terms of statistical significance (item 6a).

**Table 1:** Quality assessment summary from the modified McMaster critical review form.

Study	Study design	Items on the modified McMaster critical review form										Score, n (%)
		1	2	3a	3b	4	5	6a	6b	6c	7	
Clark et al, 2002 [40]	Case study	Y^a^	Y	Y	N^b^	Y	Y	N	Y	Y	Y	8 (80)
Forducey et al, 2003 [41]	Case study	N	Y	Y	N	Y	Y	N	N	Y	Y	6 (60)
Barlow et al, 2009 [42]	Cohort	Y	Y	Y	N	Y	Y	Y	Y	N	Y	8 (80)
Kelso et al, 2009 [43]	Case study	Y	Y	Y	N	Y	N	N	Y	Y	Y	7 (70)
Schein et al, 2010 [44]	Cohort	Y	Y	Y	N	Y	Y	Y	Y	Y	Y	9 (90)
Olsen et al, 2012 [45]	Cohort	Y	Y	Y	N	Y	N	Y	Y	Y	Y	8 (80)
Crotty et al, 2014 [46]	Cohort	Y	N	Y	N	Y	Y	N	Y	N	Y	6 (60)
Levy et al, 2015 [47]	Cohort	Y	Y	Y	N	Y	N	Y	Y	Y	Y	8 (80)
Langkamp et al, 2015 [48]	Case study	Y	Y	Y	N	Y	N	N	Y	N	Y	6 (60)
Sangelaji et al, 2017 [49]	Case study	Y	Y	Y	N	Y	Y	N	Y	N	Y	7 (70)
Portaro et al, 2018 [50]	Case study	Y	Y	N	N	Y	Y	N	Y	Y	Y	7 (70)

^a^Y=yes.

^b^N=no.

### Study Characteristics

#### Journals

A total of 11 studies were included in this review [40-50]. All of them were published in peer-reviewed journals. Each of the following journals contained 1 (9%) of the studies: Journal of Neurologic Physical Therapy [40], NeuroRehabilitation [41], International Journal of Telerehabilitation [42], Infants and Young Children [43], Assistive Technology [44], Volta Review [45], Journal of Telemedicine and Telecare [46], Journal of Rehabilitation Research and Development [47], Telemedicine Journal and E-Health [48], European Journal of Physiotherapy [49], and Disability and Health Journal [50].

#### Study Locations

A total of 7 (64%) studies were performed in the United States (2 in Oklahoma [40,41], 1 in “a large western state” [43], and 1 in each of the following states: Pennsylvania [44], Utah [45], Florida [47], and Ohio [48]). The other 4 (36%) studies were from Australia [46], New Zealand [49], Italy [50], and Canada [42].

#### Location of Study Participants

A total of 10 (91%) studies were performed with study participants in rural or remote areas, and one (9%) was conducted with participants in both a rural nursing home and in an urban community [46].

#### Year Studies Were Published

There was 1 study published in each of following years: 2002, 2003, 2010, 2012, 2014, 2017, and 2018. There were 2 studies published in 2009 and 2015.

#### Disabilities or Disability-Causing Disorders

In 5 (45%) of the studies, the specific disabilities were mentioned, 3 were DD [43,45,48] and 2 were mobile impairment (MI) [42,44]. In the other 6 (55%) studies, the cause of the disabilities were mentioned instead, 2 were stroke [40,46], 2 were MS [47,49], 1 was TBI [41], and 1 was facio-scapulo-humeral muscular dystrophy (FSHD) [50]. It must be noted that one disease may cause multiple disabilities, for instance, stroke may cause cognitive, language, and mobility impairments. Similarly, one type of disability may be caused by different medical problems. For instance, both TBI and FSHD can lead to mobility impairment. This fact made it difficult to combine numbers from these 11 studies and perform quantitative analysis.

#### Telehealth Technologies

In 9 (82%) studies, various types of VC systems were used to deliver different types of interventions. In 1 (9%) study, both a website and a VC system were used to deliver the interventions [49]. In one other (9%) study, a store-and-forward technology was used to deliver the intervention [48].

#### Type of Studies

A total of 6 studies (55%) were small-scale case studies [40,41,43,48-50], and the other 5 (45%) were cohort studies. In most of these studies, a pre- and postevaluation was performed to determine the effectiveness of the telehealth-based interventions. Questionnaires, interviews, and focus groups were used to collect data from the study participants. A few studies also included objective evaluation, such as patients’ functional level.

#### Sample Sizes

In the 6 case studies, the sample sizes were 1, 1, 4, 4, 4, and 4. Note that in one of these studies, there were 137 participants, but only 4 cases were described in detail [48]. In the 5 cohort studies, the sample sizes were 10, 26, 30, 40, and 104. In the last study, not all participants were people with disabilities [46]. The number of people with disabilities in the study was approximately 80.

#### Participant Characteristics

Not all of the studies provided the age information of their participants. In general, the participants’ age in these studies was at one of 2 extremes, either children aged 0 to 3 years or people older than 50 years. According to the information provided in the studies, the average age of the reported adult participants was approximately 60 years. Similarly, not all of the studies provided the gender information of their participants. According to the studies that did report gender for adult participants, it seems that the overall gender distribution in these 11 studies was balanced. The gender of the children in these studies was not reported. Most of the studies also did not report the race of the participants. The ones that did indicated that most of the participants were white.

#### Potential Risk of Bias

There are various types of potential risk of bias, for instance, small sample size, limited population, gender bias, being geographically limited, age bias, education bias, and racial bias. Of the 11 studies, 7 (64%) had a small sample size. As mentioned in the previous paragraph, most of the indicated study participants were white, and therefore, there was racial bias in those studies. In 5 studies (5/11, 45%), participants were either mainly male or mainly female, and therefore, there was gender bias in those studies. Although all of these studies were for people with disabilities and the participants were recruited from one or a few geographical areas, the 6 of 11 studies that were case studies have higher risk of bias because of the limited population. Only 3 studies (3/11, 27%) had good sample sizes (40, 30, 104).

These study characteristics are summarized in Table 2.

**Table 2:** Characteristics of the 11 selected studies.

Reference	Disease or disability	Sample size	Participants’ demographics	Outcome data collection method	Study location	Potential bias
Clark et al, 2002 [40]	Stroke	1	52 years, white, woman	Pre-post comparison	United States	Small sample size, racial bias, and gender bias
Forducey et al, 2003 [41]	Traumatic brain injury	1	39 years, man	Pre-post comparison	United States	Small sample size and gender bias
Barlow et al, 2009 [42]	MI^a^	10	Mean age=72.2 years, 8 women	Telehealth vs face-to-face comparison	Canada	Small sample size and gender bias
Kelso et al, 2009 [43]	DD^b^	4	Children (birth to 3 years)	Questionnaire and interview	United States	Small sample size
Schein et al, 2010 [44]	MI	40	Mean age=55 years, 36 white, 25 women	Pre-post comparison	United States	Racial bias and good sample size
Olsen et al, 2012 [45]	DD	30	Children (birth to 3 years)	Pre-post comparison	United States	Good sample size
Crotty et al, 2014 [46]	35 stroke, 10 fracture, 33 cognitive impairment, 4 joint replacement, 22 others	104	Community residents (n=61): mean age=73.4 years, 26 women. Rural nursing home patients (n=43): mean age=83 years, 30 women	Pre-post comparison, questionnaire, interview, and focus group	Australia	Good sample size
Levy et al, 2015 [47]	21 musculo-skeletal disorder, 4 MS^c^, 1 stroke	26	24 men, 18 aged 50-64 years, 8 aged >64 years	Pre-post comparison	United States	Gender bias and age bias
Langkamp et al, 2015 [48]	DD	4/137	Mean age=9.2 years, 131 white	Pre-post comparison, survey, and interview	United States	Small sample size, age bias, and racial bias
Sangelaji et al, 2017 [49]	MS	4	Ages 56, 56, 65, 75 years, all women	Pre-post comparison, interview, and questionnaire	New Zealand	Age bias and gender bias
Portaro et al, 2018 [50]	Facio-scapulo-humeral muscular dystrophy	4	4 siblings, no gender or age information	Pre-post comparison	Italy	Small sample size and limited population

^a^MI: mobile impairment.

^b^DD: developmental disability.

^c^MS: multiple sclerosis.

### Results of Individual Studies

#### Telehealth Interventions

EI for children with DD was described in 2 studies (18%) [43,45]; PT, OT, SLT, and psychology services were provided to people with stroke in 2 studies (18%) [40,46] and people with TBI in 1 study (9%) [41]; PT services were delivered to people with MS in 1 study (9%) [47]; telemonitoring, psychological consultation, and neurological and pneumological assessment services were given to 4 siblings with FSHD in 1 study (9%) [50]; typical primary care was provided to children with DD in 1 study (9%) [48]; and assessment and prescription of wheelchair and seating were provided to people with MI in 2 studies (18%) [42,44]. The duration of the interventions ranged from a few hours to 2 years.

#### Outcome Measures

In most of the studies (10, 91%), the outcome measure included participants’ (patients, care givers, local clinicians, and remote clinicians) satisfaction. In some studies, the outcome measures also included one of the following items: physical function, mental status, communication skills, self-care ability, cost and time savings, service time, number of hospital admissions, and goal attainment. The outcome measures, duration, and intervention results are summarized in Table 3.

**Table 3:** Duration, intervention, outcome measures, and intervention results for the 11 selected studies.

Reference	Duration of intervention	Interventions	Outcome measures	Results
Clark et al, 2002 [40]	17 months	PT^a^, OT^b^, SLT^c^, vocational rehabilitation, and psychological services	Mobility, self-care ability, emotion, language ability, and cost and travel savings	Patient was functionally independent in household walking and self-care; functional use of affected lower extremity for support and balance; patient could express basic needs independently, communicate complex ideas; caregiver’s mood was more positive; and cost and travel savings
Forducey et al, 2003 [41]	24 weeks	PT, OT, SLT, neuro-psychological services, and telementoring	Physical and cognitive function of patients and nursing home staff’s perception and satisfaction	Improvements in neuropsychological status and physical functioning and the telementoring program was very beneficial
Barlow et al, 2009 [42]	2 years	Wheelchair seating assessment and intervention	Patient and therapists’ satisfaction, intervention goal attainment, travel expense, therapists’ time spent in providing service, and wait time and completion time	Clients had similar satisfaction ratings to those seen F2F^d^; clients had their goals met as often as clients seen F2F; travel cost savings; rural therapists spent more time in preparation and follow-up; and clients had shorter wait times for assessment than rural F2F clients
Kelso et al, 2009 [43]	1 month for 2 families, 3 months for the other 2 families	EI^e^ (SLT, OT, and PT)	Parental satisfaction, usability of the system, interventionists’ feedback, and cost and travel savings	Videoconferencing-based tele-EI system is both usable and satisfactory to most participants; parents and therapists experienced technical problems; and cost savings for delivering EI via telehealth
Schein et al, 2010 [44]	88 min on average	Assessment and prescription of wheelchair and seating	Users’ satisfaction, comfort and time and cost savings	A high level of patient satisfaction and saved money and time
Olsen et al, 2012 [45]	1 year	EI, home visits, and coaching model	Cost savings, participants’ rating, and provider and family satisfaction	Cost savings and increased availability of services from specialists; parents’ comfort with technical skills was high; provider’s ratings of comfort with the telehealth experience were high; parents were satisfied with each visit modality; most providers (79%) were satisfied with the telehealth experience; and telehealth removed time and travel barriers and increased availability of qualified personnel
Crotty et al, 2014 [46]	Up to 8 weeks	Coaching model, feedback and homework for the patient, SLT, OT, PT, and medical reviews	Participants’ satisfaction, goal attainment, number of home visits, service time, travel time; cognitive impairment, mood, quality of life, and functional level and perceived ease of technology use	Participants achieved 75% of the goals; high levels of satisfaction; a 50% reduction in home visits by staff; speech therapists doubled occasions of services and direct patient contact time but halved their travel time; patients achieved >50% of their goals; most patients achieved their anticipated or better outcome; telehealth was acceptable and perceived positively by older people; and in approximately 2/3 cases, clinicians were equally satisfied with telehealth compared with F2F sessions
Levy et al, 2015 [47]	On average 99 days	PT	Functional level, quality of life, and satisfaction	Significant improvement in most outcome measures; 96% of patients were satisfied with the telehealth experience; and avoided travel miles, driving time, and travel reimbursement
Langkamp et al, 2015 [48]	1 year	Connection to primary doctor	Parents’ satisfaction, school staff's satisfaction and comfort with the program, and participating practice members’ experience with the program	Most parents had a high level of satisfaction with the program; parents were satisfied with the care their child received; school staff noticed benefits of telehealth; and participating providers agreed to continue the participation
Sangelaji et al, 2017 [49]	24 weeks	12 weeks Web-based physiotherapy followed by 12 weeks behavioral change intervention	Participants’ feedback, physical activity, body function and composition; quality of life, fatigue, and mental status	Intervention was not effective for the participants; accepted telehealth practice; overall dissatisfaction with using the activity monitors; and both positive and negative aspects of website use
Portaro et al, 2018 [50]	6 months	Telemonitoring, psychological consultation, neurological, and pneumological assessment	Number of hospital admissions, patients’ satisfaction, the clinical impact, and quality of life	Reduced hospital admissions; patients had a mild improvement in emotional and mood status; body mass index remained stable; patients developed better skills to solve problems; no change on caregiver burden; and reasonable level of satisfaction

^a^PT: physical therapy.

^b^OT: occupational therapy.

^c^SLT: speech and language therapy.

^d^F2F: face-to-face.

^e^EI: early intervention.

## Discussion

### Principal Findings

Long-term and highly skilled therapists in various fields (such as PT, OT, SLT, and psychotherapy) are not available in underserved areas, including rural areas, remote areas, some poor urban communities, and developing countries. Telehealth may be a viable approach for delivering intervention digitally to people with disabilities in such underserved areas.

This systematic review showed that most patients had a positive opinion regarding digital intervention delivered via telehealth. Most of them had reasonable levels of satisfaction; some of them had functional improvement in motor performance, language ability, and self-care skills. Their mental status and quality of life showed improvement in some studies. In addition, telehealth made it possible for them to access desired interventions and saved them time and money.

A few studies included in this review provided services and evaluated the situations of family caregivers [40,50]. The results indicated that these caregivers were helped by the digital intervention (such as psychotherapy and communication skills) and that caregivers were satisfied with the intervention delivered to patients via telehealth.

Some studies also assessed the local and remote care providers’ experience with participating in the telehealth-based intervention [46,48]. Overall, these care providers were generally satisfied with this digital intervention delivery approach as it provided intervention results comparable with face-to-face visits, increased patient contact time, and reduced travel time and costs.

The majority (6, 54%) of the 11 studies were small-scale case studies, and the rest were relatively larger-scale cohort studies. None of them were randomized controlled trials. Most of these studies used pre-post evaluation, questionnaire, and interview to determine the outcome of the intervention. They did not offer comparison with the outcomes of traditional face-to-face intervention.

For some specific interventions, such as EI, it is known that for children it is beneficial to be delivered within the child’s natural environment and to use daily activities with familiar people. In this case, digital intervention via telehealth might be the only plausible approach for delivering EI to children in underserved areas at a specific time and frequency.

In most of these studies, the telehealth technology was VC for synchronized intervention, in which all parties (patients, caregivers, local care providers, and the remote care team) could interact in real time. This is desired in most cases. In some circumstances, asynchronous telehealth may be superior to synchronized communication or traditional in-office visits [48], as children with DD may not cooperate when a doctor is observing. In a store-and-forward mode, children may not have the stress, and they are more likely to cooperate when having a medical exam done by a school staff they know.

### Comparison With Previous Studies

There have been some other systematic reviews of telehealth or telerehabilitation in general, but these typically only focused on a specific disease, a specific age group, a specific type of outcome, or a specific geographical area [34,51]. Our systematic review covered studies performed all over the world and with all types of disabilities and disability-causes diseases. Our study included both synchronous and asynchronous interventions with all ages. The results of our systematic review and narrative analysis are consistent with those of other reviews [34].

In 2000, a systematic review of studies of patient satisfaction with telehealth reported findings in 32 studies conducted worldwide and published between 1966 and 1998 [52]. It pointed out that although all studies reported a good level of patient satisfaction, qualitative analyses determined methodological problems with all the published work, such as low sample sizes and problematic study design, which limited the generalizability of the findings in those studies [52].

In 2003, a keyword search (“telehealth, telemedicine, or telerehabilitation”) in the literature returned mostly pilot studies, case studies, and feasibility studies [53]. The situation was not significantly improved in 2007 as many of the identified studies still had limitations in study design, small sample size, and no comparison with face-to-face intervention [54].

Our systematic review covered studies published between 2003 and 2018. Comparing our results with those of the previous studies, we can see that the research studies in telehealth have not significantly improved in the past two decades. The studies reviewed in this project still suffered from the same issues: small sample size and lack of comparison with face-to-face intervention.

Some people believe that the small sample size in telehealth studies is related to the availability of technology or familiarity with the ICT used in telehealth [55,56]. The wide adoption of the internet and smart mobile devices in recent years indicate that the availability of technology is not an issue anymore. A few studies included in this review evaluated participants’ familiarity with technology and its impact on the outcomes of the intervention [45,46]. The general conclusion was that older people were less familiar with technology [57] but that age and previous experience with ICT were not barriers to digital intervention via telehealth if technology training was provided before the intervention. However, it is possible that familiarity with technology might impact study participant recruitment [46].

### Limitations

This systematic review contains several limitations. The keyword search did not use a controlled vocabulary (eg, Medical Subject Headings). The inclusion criterion may have excluded studies that describe digital telehealth intervention for people with disabilities in underserved areas but do not contain the exact keywords we used. Moreover, only studies with full text written in English were included in the sample, which excluded articles in non-English journals. In addition, only peer-reviewed studies published in scholarly journals and conference proceedings were included in this study; therefore, articles published in gray literature were excluded.

Several concerns regarding the selected studies and outcomes limited the overall findings of this study. The included studies had highly heterogeneous designs and used various methods to measure the outcomes of digital interventions. Therefore, it was not feasible to conduct meta-analysis or explore the impact of these studies as a group. In addition, some studies did not include clear descriptions of the recruitment process. The studies included were at risk of selection bias, and on the individual study level, there was a lack of information about potential confounding factors such as age, gender, and educational level, which possibly could have affected self-assessed outcome.

We acknowledge that the number of included studies is small, reflecting the current state of published literature relating to digital telehealth intervention for people with disabilities in underserved areas. This review may serve as a checkpoint for the development of more, larger-scale, and higher-quality digital telehealth intervention studies for people with disabilities in underserved areas by researchers in the future. The findings from this study itself are limited by the small number of studies that met the inclusion criteria and the small sample sizes involved in each study. Therefore, although the obtained results were mainly positive, because of the small sample size, they may be considered only proof of concept instead of solid and generalizable conclusions.

This study did not include a regular telephone-based intervention. This is one of the feasible approaches for providing intervention to people in underserved areas. However, in many cases, rehabilitation intervention requires demonstration of proper procedure, and that is very difficult if not impossible via a regular telephone conversation. Considering that many people in the underserved areas, including people in developing countries, have mobile phones instead of land phones and the rate of ownership is still increasing, it is believed that many people in underserved areas can have VC for telehealth-based interventions, and therefore, we believe this decision would not have led to missing any important studies.

There are several ongoing studies in this field as well as study protocols with larger sample size [58-60]. These may generate better and more convincing results in the near future.

## Abbreviations

CP: cerebral palsy
DD: developmental disability
EI: early intervention
FSHD: facio-scapulo-humeral muscular dystrophy
ICT: information and communication technology
IVR: interactive voice response
MI: mobile impairment
MS: multiple sclerosis
OT: occupational therapy
PT: physical therapy
SLT: speech language therapy
TBI: traumatic brain injury
VC: videoconferencing
